# The influence of journal submission guidelines on authors’ reporting of statistics and use of open research practices: Five years later

**DOI:** 10.3758/s13428-022-01993-3

**Published:** 2022-10-17

**Authors:** David Giofrè, Ingrid Boedker, Geoff Cumming, Carlotta Rivella, Patrizio Tressoldi

**Affiliations:** 1 DISFOR, University of Genoa, Corso Andrea Podestà, 2, Genoa, Italy; 2 School of Psychology and Public Health, La Trobe University, Melbourne, Australia; 3 Studium Patavinum, University of Padua, Padua, Italy

**Keywords:** Statistical practices, Open science practices, Author submission guidelines

## Abstract

Changes in statistical practices and reporting have been documented by Giofrè et al. *PLOS ONE 12*(4), e0175583 (2017), who investigated ten statistical and open practices in two high-ranking journals (*Psychological Science* [PS] and *Journal of Experimental Psychology-General* [JEPG]): null hypothesis significance testing; confidence or credible intervals; meta-analysis of the results of multiple experiments; confidence interval interpretation; effect size interpretation; sample size determination; data exclusion; data availability; materials availability; and preregistered design and analysis plan. The investigation was based on an analysis of all papers published in these journals between 2013 and 2015. The aim of the present study was to follow up changes in both PS and JEPG in subsequent years, from 2016 to 2020, adding code availability as a further open practice. We found improvement in most practices, with some exceptions (i.e., confidence interval interpretation and meta-analysis). Despite these positive changes, our results indicate a need for further improvements in statistical practices and adoption of open practices.

**Supplementary Information:**

The online version contains supplementary material available at 10.3758/s13428-022-01993-3.

Over the last decade, many published findings have been found not to be fully replicable (Camerer et al., 2018; Open Science Collaboration, 2015). This observation has led scientists, funders, research users, and policymakers to investigate the reasons behind the absence of replicability (e.g., Goodman et al., 2016). Several factors have been considered, including the presence of questionable research practices, statistical problems (including low statistical power), and barriers to data sharing, including a lack of encouragement and training from journals and institutions to publicly share data (Agnoli et al., 2017; Houtkoop et al., 2018; Szucs & Ioannidis, 2017; Tressoldi & Giofrè, 2015). To overcome these problems, several journals have introduced new statistical guidelines, which encompass new statistical standards and other research practices, including providing raw data and other materials online.

One influential approach, advocated by Cumming (2013, 2014), has been use of the so-called “New Statistics,” a set of best practices for making inferences from data (Calin-Jageman & Cumming, 2019). Included are the need: i) to address uncertainty in all statistical conclusions, seeking ways to quantify, visualize, and interpret the potential for error (e.g., by including confidence intervals or Bayesian credible intervals); and ii) to pursue replication, and use quantitative methods to synthesize across different data sets (e.g., performing meta-analyses or using informed priors). More broadly, Open Science practices are designed to enhance the trustworthiness of research results. In order to increase the uptake of these practices, some journals have explicitly recommended their use in the journal requirements/instructions for authors, which has had an impact in changing researchers’ behavior and increasing openness, data sharing, and data availability (Giofrè et al., 2017; Kidwell, Lazarević, Baranski, Hardwicke, Piechowski, Falkenberg et al., 2016).

The changes in statistical practices and reporting have been documented by Giofrè et al. (2017), who investigated the statistical practices and open practices in two high-ranking journals (*Psychological Science* [PS] and *Journal of Experimental Psychology, General* [JEPG]). The investigation was based on an analysis of all papers published in these journals between 2013 and 2015. Results showed that the introduction of new statistical guidelines, in particular for PS, was followed by an improvement in most statistical practices, for example, reporting confidence or credible intervals, and reporting more information about the sample (e.g., sample size determination and data exclusion practices). Concerning Open Practices, the authors found that the inclusion of new guidelines was beneficial, for example, in increasing the number of papers providing raw data and additional materials. However, the authors also found some limits related to the introduction of these new practices and guidelines.

Since 2015, more than 2000 journals have implemented policies that align with one or more of the standards covered in the Transparency and Openness Promotion (TOP) Guidelines (Nosek et al., 2015) in their journals, and a general improvement in statistical practices has also been documented in recent years by Blanca et al. (2018). However, the introduction of new practices might, despite these being beneficial, have led to only superficial changes. Giofrè et al. (2017) claimed that from 2015 to 2017, PS and JEPG were still over-reliant on an NHST approach (i.e., an approach overly focused on the *p* value and not on the interpretation of the effect size or using a different approach, such as Bayesian). And now, although researchers may be reporting more information, they may still be interpreting and basing their conclusions using a traditional NHST approach, which has been criticized on several occasions (Tressoldi et al., 2013).

Giofrè et al. (2017) also found that, although the use of preregistration had been strongly recommended by PS in the new guidelines, it was scarcely used in both PS and JEPG. By 2018, proponents of preregistration were still calling it an emerging practice (Nosek et al., 2018). Because they were relatively new when the previous research was conducted, it is worth investigating whether preregistrations have become more prevalent in recent years. Finally, since the previous research was limited to only 3 years, the question of the general long-term effects of the changes remains to be fully addressed.

As far as changes in the guidelines in recent years are concerned, the 2020 edition (7th edn.) of the Publication Manual has recently been released (American Psychological Association, 2020). However, it will take time to see any resulting changes implemented at a journal-specific level. The past and present research concerns the instructions to authors given by the editors of journals, not the APA. This difference is crucial, as previous research has shown that simply referring authors to an external source (such as APA) may not be as effective as publishing journal-specific guidelines (Giofrè et al., 2017).

PS recently updated its guidelines on August 18, 2021, as can be found here: https://www.psychologicalscience.org/publications/psychological_science/ps-submissions (accessed September 2021). These guidelines remain very similar to the ones published in 2015, with some parts reworded. For example, in the previous version, PS asked authors to provide a rationale for the sample size; in 2021 they specified where in the manuscript this should go. A blurb was added about replicability being not the only consideration but an important one; authors are also now encouraged to use graphical presentations to show how data are distributed and to include information about sample sizes in the manuscript’s abstract.

Similarly, JEPG updated its guidelines from July 1, 2021, as can be found here: https://www.apa.org/pubs/journals/xge/ (accessed September 2021). Recent changes have included requiring authors to state whether data are publicly available and where they can be accessed; whether or not work has been preregistered. Time will tell how these specific guidelines will affect transparency in the long run, but for the purposes of the present study, in the period from 2016 to 2020, these journal-specific guidelines, or in the case of JEPG, a lack thereof, had not changed markedly.

The aim of the present study is to document any changes in authors’ statistical practices in PS and JEPG from 2016 to 2020. We investigated the previously studied practices, and added one more: the availability of statistical code, or script (typically R code). Subsequent to the publication of the original report (Giofrè et al., 2017), many psychological researchers have started to learn dynamic writings such as Rmarkdown, which is useful for fully reproducing the results of statistical analyses. Making statistical scripts available has been endorsed recently by Goldacre and co-authors (Goldacre et al., 2019) and is included in the TOP Guidelines (https://www.cos.io/initiatives/top-guidelines).

We hypothesized a continuation of the findings of Giofrè et al. (2017; see also https://osf.io/preprints/metaarxiv/e6whp). We hypothesized that improvements in statistical practices and open practices in PS, which changed its journal submission guidelines in 2014, would be larger than the improvements in JEPG, which only referred to third-party guidelines. While we did expect to see an increase in the adoption of the open practices, building on those observed in Giofrè et al. (2017), we expected a much smaller increase, in both journals, in the interpretation of results based on confidence intervals and effect sizes of the main outcomes. Instead, we hypothesized that a large number of papers would still rely mainly on dichotomous interpretations (e.g., there is/there is not a difference, a correlation, or a superiority of model X with respect to model Y, etc.) instead of magnitude interpretations (e.g., there is a strong/small difference, correlation, etc.).

## Materials and methods

The database for the present study is posted on the online open access repository https://osf.io/q2bn9/.

### Inclusion and exclusion criteria

All papers published in PS and JEPG between January 1, 2016 and December 31, 2020, were considered. Only primary empirical papers reporting data from one or more empirical studies and their online supplemental materials, when available, were considered. Papers reporting only meta-analysis, narrative reviews, simulation, comments, or theoretical studies were excluded. Papers reporting a meta-analysis of results from multiple earlier papers were excluded, whereas those reporting a meta-analysis of multiple findings reported in that same paper were included. These inclusion and exclusion criteria are identical to those used by Giofrè et al. (2017).

### Scoring procedure and method (see also Table 1)

The list of practices reported below was adapted from Cumming (2013), the Open Science Framework badges scheme (Kidwell et al., 2016; Blohowiak et al., 2018), and the TOP Guidelines (Open Science Foundation, https://osf.io/). The scoring procedure and method is identical to the procedure adopted by Giofrè et al. (2017, p.4) except for the inclusion of a new practice (the 11th).

**Table 1:** Number of papers included in the two journals, from 2016 to 2020, and total

Publication Year	2016	2017	2018	2019	2020	Total
JEPG	115	110	109	130	143	607
PS	146	153	157	137	119	712

“Published journal papers, and online supplemental material when available, were considered. A single occurrence of a practice anywhere in the paper was sufficient for a coding of 1, indicating that this practice had been used, of 0, if had not been used, and “NA” if not applicable. Papers were examined for the following eleven practices:

1. NHST. A *p* value was reported, whether exact (PE; e.g., *p* = 0.036) or relative (PA; e.g., *p* < 0.05).
2. CI. A confidence interval or credible interval (in the case of Bayesian statistics) was reported for either a standardized or unstandardized measure.
3. MA. Meta-analysis of multiple related results included in the paper was reported. We only included papers with more than one experiment related to the same empirical question.
4. CI_interp. A confidence interval was referred to and used to inform the discussion or interpretation of the results. For example, this would include a paper explicitly mentioning the width or the precision of the CI, a comparison between two or more CIs, or an overlapping between two intervals.
5. ES_interp. An effect size, either standardized or unstandardized, was referred to in the discussion or interpretation of the results. We considered ‘effect size’ in the broad sense, including means, differences between means, percentages, and correlations, as well as Cohen’s *d*, *R*^2^, and *η*^2^. Papers were considered which included not only a dichotomous difference vs. no difference approach, but also those referring to the magnitude of the effect (e.g., small, large, strong etc.) or to the amount of explained variance.
6. Sample_size. The authors had a statistical rationale for how sample size(s) was determined. For example, a prospective power analysis – based on previous research, or on an estimated effect size – had been conducted. We excluded papers where the sample size was left to chance, e.g., “Sample size was determined by signup,” or, “Sample size was determined by participants’ availability."
7. Data_excl. Authors explicitly declare if and which stopping rule was adopted or the criteria used to exclude data/participants.
8. Data. The paper carried the Open Data badge (see below), or stated where the data were available or how they could be obtained. We used a very lenient approach, including all papers mentioning that data were available (e.g., data are available upon request).
9. Materials. The paper carried the Open Materials badge, or stated where details of the experimental materials and procedure could be obtained. We used a very lenient approach, including all papers mentioning that materials were available (e.g., materials are available upon request).
10. Preregistered. The paper carried the Preregistered badge, or stated where a preregistered plan had been lodged in advance of data collection. Papers in this category typically included information about the number of the preregistration or where the preregistration is available.
11. Code. The paper provided, in the paper, the supplementary materials, or the preregistration, the computer code or syntax (e.g., R code) needed to reproduce the analyses. We used a very lenient approach; for example, we did not check if the code was sufficient to successfully replicate all of the analyses presented in the paper.

The three badges (i.e., Data, Materials and Preregistration) are described in detail by the Center for Open Science (tiny.cc/badges; accessed September 2021). For PS, these badges were certified by an “earned badge” from the Open Science Framework (https://osf.io/tvyxz/wiki/home; accessed September 2021).

The Open code badge is described here: https://www.comses.net/resources/open-code-badge (accessed October 2022).

For JEPG, badges are not available, but we coded whether or not authors clearly indicated how to obtain the data, materials, and code.”

### Data analysis

#### Scoring method and reliability

The first level of scoring method and reliability followed exactly the same rules as in the original Giofrè et al. (2017) paper. These procedures are reported below.

Papers were examined for the presence of each of the 11 practices. For each, the score could be 1 if present, 0 if absent, and “NA” if not applicable. The total number of included papers for each journal, from 2016 to 2020, are reported in Table 1.

Each paper was independently scored by one of the authors (i.e., since the number of papers was particularly high, it was impossible for the same author to score all papers, therefore several scorers were necessary). The authors are experienced researchers with good knowledge of the statistics examined. Second, a random sample of 137 papers, being roughly 10% of the whole set of papers, was scored independently by a second judge to test inter-rater reliability. The overall agreement was 96%, with discrepancies resolved by discussion.

Only descriptive statistics are reported, given that they refer to the whole population of papers. For each of the 11 practices analyzed, the number of papers including a practice are expressed as a percentage of the total number of papers. It is also worth mentioning that for ES_Interpr and for CI_Interpr the denominator corresponded the total number of all included papers, not the percentage of papers using an ES or a CI. The checklist for study examination is presented in Table 2 and is identical to the procedure adopted by Giofrè et al. (2017, see Table 1) except for the inclusion of a new practice (the 11th).

**Table 2:** Checklist for study examination, related to the core experiment(s) and not to pilot ones

Paper ID:			
	Value	Labels	Criteria for ‘1’ response
Statistics			
1-Null hypothesis significance testing	PE (*p* exact)/PA (*p* relative)/0	NHST	At least one *p* value is reported; format: exact: e.g., *p* = 0.35; approximated: e.g., *p* < 0.05. Add other different types of statistics if available: e.g., Bayes; SEM, etc.
2-Confidence intervals	1/0	CI	At least one is reported.
Statistical approach			
3-Meta-analysis of reported data	1/0/NA	MA	Authors meta-analyze results (applicable only where the results of more than one experiment are reported).
4-Confidence intervals interpretation	1/0	CI_Interpr	Authors explicitly refer to CIs in the comments and/or discussion of the results, e.g., *confidence intervals remained narrow enough…*
5-Standaridzed or unstandardized effect size interpretation	1/0	ES_Interpr	Authors explicitly refer to *ES*s in the comments and/or discussion of the results, e.g., *The effect size for the difference (11.94 percentage points) was large; effect sizes were moderate for comparisons with the low-intensity shock conditions.*
Research practice disclosures			
6-Sample size determination	1/0	Sample_size	Authors explicitly clarify how they determined the sample size(s), i.e., prospective power estimate; expected effect size.
7-Data exclusion	1/0	Data_excl	Authors explicitly declare if and which stopping rule was adopted or the criteria used to exclude data/participants.
Open practices			
8-Data availability	1/0	Data	Authors explicitly give information on how the data may be obtained, e.g., posted in a repository; author e-mail, etc.
9-Materials availability	1/0	Mater	Authors explicitly give information on how to obtain the materials, equipment and/or software used in the study.
10-Preregistered design & analysis plan	1/0	Prereg	Authors explicitly declare where the study was preregistered or that it is a Registered Report.
11-Code availability	1/0	Code	Authors provide the statistical code in the paper, supplementary material, or preregistration.

### Changes from the preregistration

We decided to use percentages rather than proportions. The two are equivalent, but percentages are more commonly reported. As for the Sample_size determination criterion, in which authors clarified how they determined the sample size, on several occasions authors used very vague statements (e.g., the sample size was chosen based on previous research on this topic). Therefore, we also decided to check how many papers used a more stringent criterion, that is if authors determined their sample size using a power analysis (Pwr_analysis). Also in this case, we used a very lenient approach, any mention to a power analysis (even on a single experiment rather than on all experiments), qualified to achieve this criterion (see additional results).

## Results

**Fig. 1:**
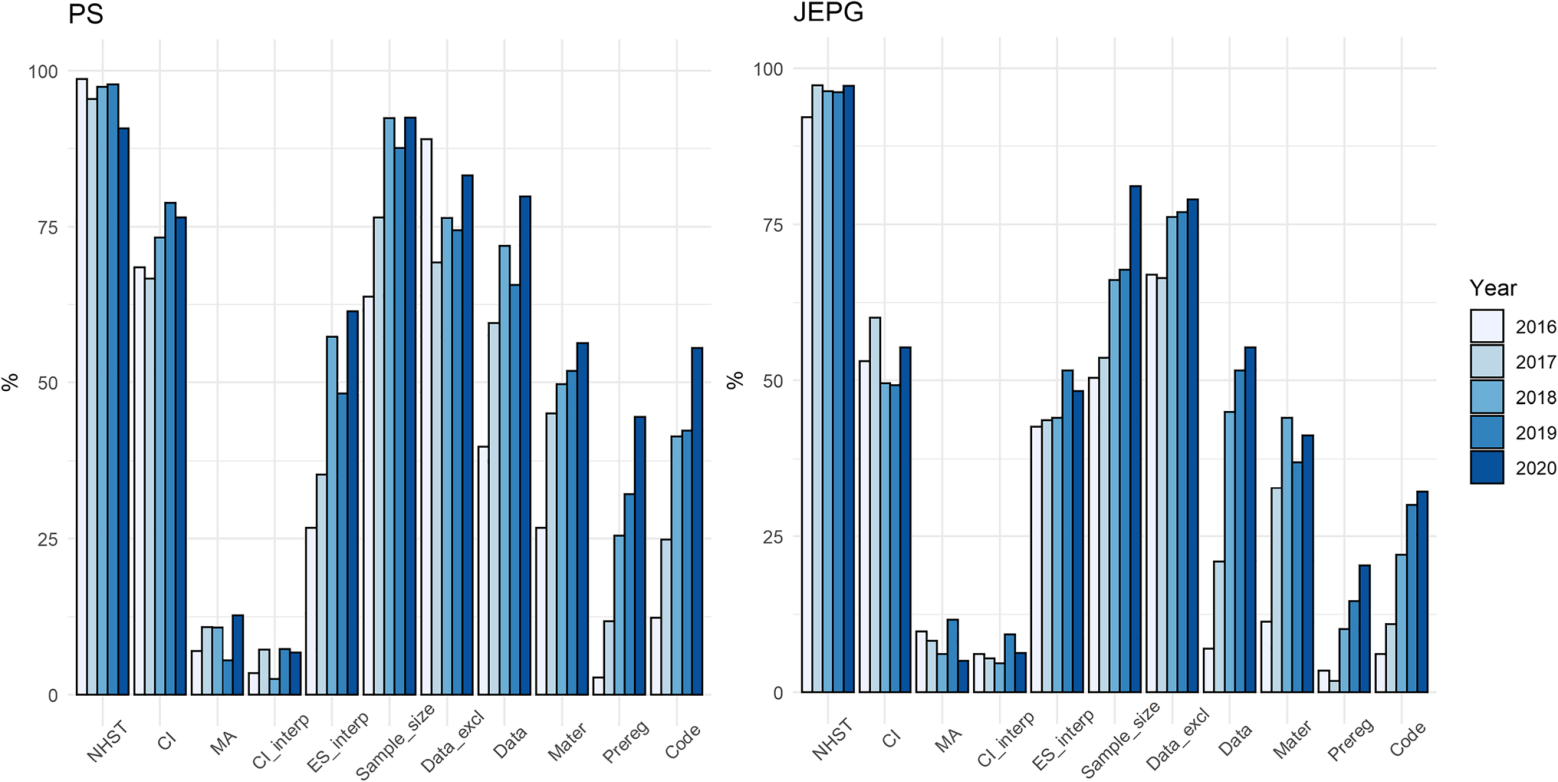
Percentages of the ten practices, from 2016 to 2020 in *Psychological Science* and in the *Journal of Experimental Psychology*: *General. *See Table 2 for definitions of the abbreviations

Table 3 and Fig. 1 report the percentage of papers using a practice, for each journal for each year. Note that most of the studies were original investigations; only 1.9% were replications of previous studies.

**Table 3:** Percentages of the 11 practices, from 2016 to 2020, in PS and the JEPG

Publication year	PS					JEPG				
	2016	2017	2018	2019	2020	2016	2017	2018	2019	2020
NHST	98.63	95.42	97.45	97.81	90.76	92.17	97.27	96.33	96.15	97.20
CI	68.49	66.67	73.25	78.83	76.47	53.04	60.00	49.54	49.23	55.24
MA	6.98	10.84	10.71	5.48	12.73	9.76	8.24	6.10	11.63	5.05
CI_interp	3.42	7.19	2.55	7.30	6.72	6.09	5.45	4.59	9.23	6.29
ES_interp	26.71	35.29	57.32	48.18	61.34	42.61	43.64	44.04	51.54	48.25
Sample_size	63.70	76.47	92.36	87.59	92.44	50.43	53.64	66.06	67.69	81.12
Data_excl	89.04	69.28	76.43	74.45	83.19	66.96	66.36	76.15	76.92	79.02
Data	39.73	59.48	71.97	65.69	79.83	6.96	20.91	44.95	51.54	55.24
Mater	26.71	45.10	49.68	51.82	56.30	11.30	32.73	44.04	36.92	41.26
Prereg	2.74	11.76	25.48	32.12	44.54	3.48	1.82	10.09	14.62	20.28
Code	12.33	24.84	41.40	42.34	55.46	6.09	10.91	22.02	30.00	32.17

See Table 2 for definitions of the abbreviations

Table 3, and Figs. 1 and S1 report that a NHST approach was prevalent in both journals (well above 90%), with some year-by-year variation. This result is in line with our expectations. While these percentages are clearly very high, at the same time, it is hard to determine if this constitutes an overreliance on an NHST approach. We found that a minority of papers (less than 10%), coded as NHST, used an approximated *p* value (Table 4). This result is encouraging, showing a decline compared to the past (Giofrè et al., 2017), when percentages of over 10% and close to 20% of papers used an approximated *p* value in the two journals. In line with our expectations, in general, exact *p* values were used a little more frequently in PS, which used journal-specific guidelines, than JEPG, which referred to third-party guidelines.

**Table 4:** Percentages of approximated and exact *p* values, from 2016 to 2020, in PS and the JEPG

Publication Year	PS					JEPG				
	2016	2017	2018	2019	2020	2016	2017	2018	2019	2020
PA	6.94	2.05	3.27	0.00	7.41	8.49	5.61	7.62	8.00	7.19
PE	93.06	97.95	96.73	100.00	92.59	91.51	94.39	92.38	92.00	92.81

*PA* approximated *p* value, *PE* exact *p* value

For the presence of CIs, we previously reported, and show in Fig. S2 that the percentage of papers including at least one CI in 2015 was 70% for PS and 52% for JEPG, following a dramatic increase from 2012 to 2015. However, percentages from 2015 to 2020 in both journals remained fairly constant (Table 3 and Figs. 1 and S2). Higher rates were evident in PS than JEPG.

Concerning meta-analysis application (i.e., a meta-analysis when multiple studies on the same outcome were performed in one paper), we found that in both journals, percentages were extremely low (Table 3, Figs. 1 and S3). It is also worth mentioning that those percentages were not very different from those observed by Giofrè et al. (2017), with very minor changes. It is also worth mentioning that the denominator of this category only included papers for which it was possible to calculate a meta-analysis of multiple findings reported in the same paper, meaning that those percentages are only reflecting papers for which it was possible to perform a meta-analysis (see also Table 5).

**Table 5:** Number of papers calculating a meta-analysis, from 2016 to 2020, in PS and the JEPG

Publication Year	PS					JEPG				
	2016	2017	2018	2019	2020	2016	2017	2018	2019	2020
0	80	74	75	69	48	74	78	77	76	94
1	6	9	9	4	7	8	7	5	10	5
NA	60	70	73	64	64	33	25	27	44	44
Total	146	153	157	137	119	115	110	109	130	143

*NA* not possible to calculate a meta-analysis of multiple findings reported in the same paper

The rate of CI_interpretation in both journals is extremely low, and has not improved over time (Giofrè et al., 2017). It is also worth mentioning that these percentages were calculated on the full set of included papers (i.e., after reviews, meta-analyses, comments, etc., are excluded). This is extremely disappointing and important, highlighting that most authors do not address the precision of their results (Table 3, Figs. 1 and S4).

For ES_interpretation, we found a considerable and welcome improvement in the two journals from 2013 to 2015, but little change over the last few years and still only around 50% of papers, at most, include any interpretation of effect sizes (Table 3, Figs. 1 and S5). It is also worth mentioning that these percentages were calculated on the full set of included papers.

The percentage of papers including some sample size explanation is presented in Table 3 and Fig. 1. This percentage has steadily grown from 2013 to 2020 in both journals. It is somewhat higher for PS (Fig. S6), as expected. We investigated this issue further by calculating the percentage of papers reporting an a priori power analysis (see additional results below).

As for data exclusion, we found that the percentage of papers using this criterion steadily increased throughout the years (Fig. 1). It is encouraging that close to 80% of papers in the two journals reported this practice (Fig. S7).

Concerning Open Practices, results are encouraging (Table 3, Fig. 1). The percentage of papers reporting open Data (Fig. S8), open Materials (Fig. S9), and Preregistrations (Fig. S10) and Code (S11) has sharply increased and shows a positive trend in both journals (percentages were much lower in the past; see Giofrè et al., 2017). Figures S8, S9, S10 and S11 show that percentages were considerably higher in PS, in which authors receive a badge if they comply with the open practice, than in JEPG. This difference was particularly marked for Preregistration.

For the reporting of the code, a newly introduced practice, we found a sharp improvement in the last 5 years, with higher percentages in PS than JEPG (Fig. S11).

### Additional results

**Fig. 2:**
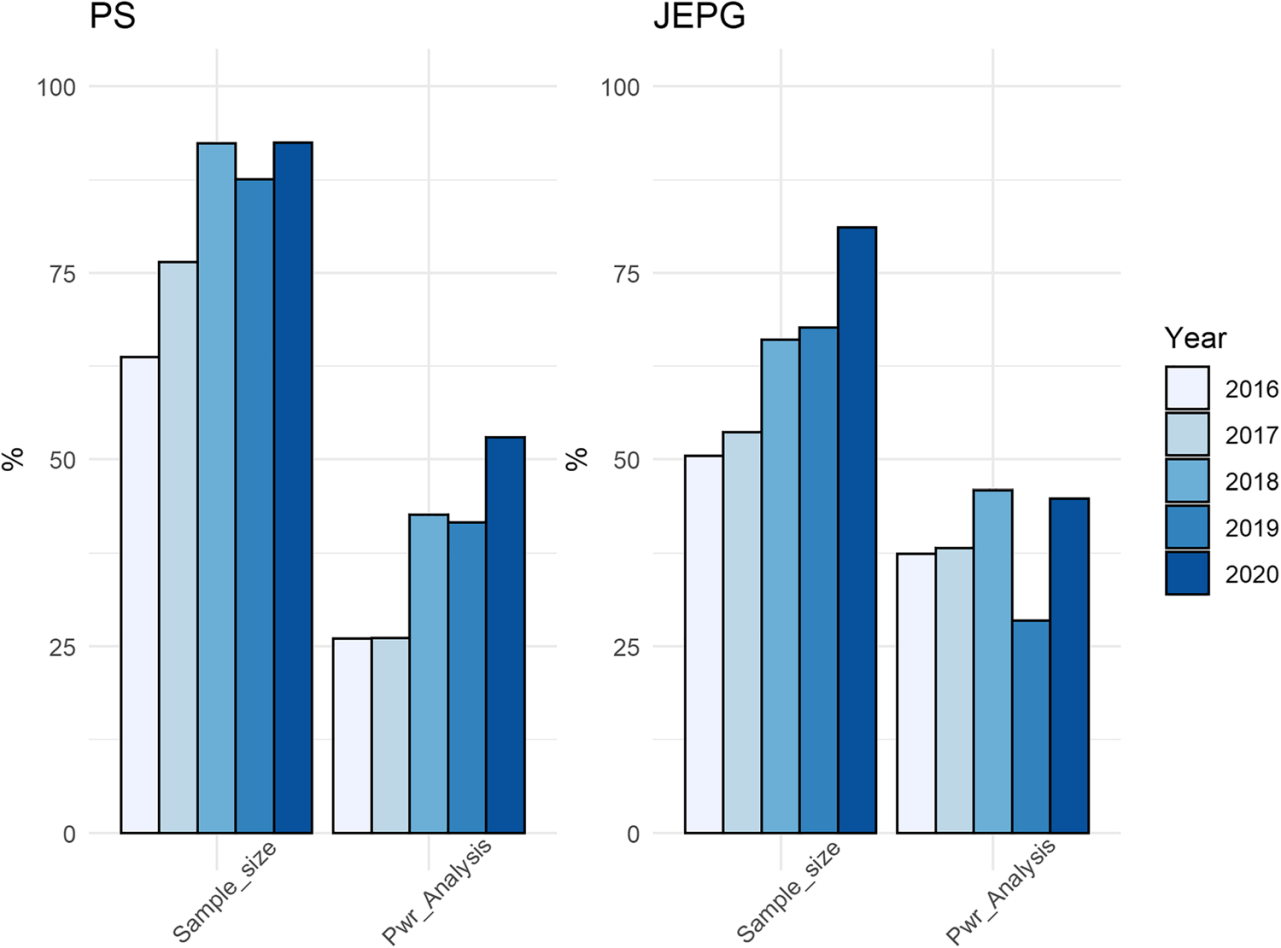
Percentages of papers reporting sample size determination and papers reporting an a priori power analysis in *Psychological Science* and in the *Journal of Experimental Psychology: General. *Sample_size = Sample size determination; Pwr_analysis = Power analysis

We added a practice, which was not included in the preregistration: reporting an a priori power analysis. It is worth mentioning that papers performing a power analysis were also coded for sample size determination. It is also worth mentioning that both percentages (for Sample_size and Pwr_Analyses) were calculated on the full set of included papers (i.e., after reviews, meta-analyses, comments, etc., are excluded). As expected, the percentage of papers reporting an a priori power analysis was much lower than the percentage of papers reporting on sample size determination. The pattern was similar in the two journals (Table 6; Fig. 2).

**Table 6:** Percentages of papers calculating a power analysis, from 2016 to 2020, in PS and the JEPG

Publication Year	PS					JEPG				
	2016	2017	2018	2019	2020	2016	2017	2018	2019	2020
Power analysis	26.03	26.14	42.68	41.61	52.94	37.39	38.18	45.87	28.46	44.76

## Discussion

The aim of the present study was to follow up changes in PS and JEPG in subsequent years, from 2016 to 2020, adding code availability as a further open practice.

The so-called replicability crisis originated from observations that findings in a variety of areas of science failed to replicate (Open Science Collaboration, 2015). Replications are of fundamental importance for scientific development (Lindsay, 2015). Despite this importance, only a tiny percentage of papers published in PS and JEPG is a replication. This might be in part due to the reluctance of authors to perform replications in favor of other types of works, so initiatives directed to increase the number of replications in top journals are probably needed.

Concerning the use of NHST, we confirmed that, in line with Giofrè et al. (2017), there is still an overreliance on interpreting results based on NHST. Despite this, some positive results are also emerging: For example, the use of CIs and ES interpretation has increased notably in both journals. Growth in these two practices is higher in PS, in which journal-specific guidelines have explicitly encouraged the authors in this direction. Even when CIs were reported, however, we found authors tend to neglect their importance, avoiding commenting on or interpreting those intervals. We found that the rate of CI_interpretation in both journals is very low, and has not improved from past years (Giofrè et al., 2017). This is extremely important, and highlights the fact that most authors may fail to understand the problem of precision. For example, even in presence of a large effect size, if intervals are very wide, the real effect can fall anywhere within this interval (typically but not exclusively within 95%). This has a crucial impact and is extremely important, for example, for a full interpretation of the results, for the calculation of statistical power, and for meta-analyses.

Concerning sample size determination and data exclusion, we found a sharp improvement in both journals. We were very pleased to find that most of the papers in PS, and a large majority of papers in JEPG, are now providing some explanation of how the sample size was determined. Since statistical power is often neglected (Tressoldi & Giofrè, 2015), we decided to investigate this issue further by also coding whether papers reported a prospective power analysis. In both journals, as Fig. 2 illustrates, only a minority of papers making some statement about sample size determination reported an a priori power analysis. It is also worth mentioning that, by definition, small sample size usually gives very long CIs, so great uncertainty. For this reason, if an ES from a very small study is used in a future power analysis, it is very highly uncertain how relevant or helpful the result is, for planning future studies. In other words, a power analysis is very highly dependent on the value entered for ES, and relying on the estimated ES from a small study can give more or less any power result.

As we mentioned previously in relation to confidence intervals, authors seem not to be particularly aware of the problem of precision. One easy and economical way of reducing uncertainty is to perform a meta-analysis of multiple findings reported in the same paper. Tables 3 and 5 show that only a small minority of papers performed a meta-analysis when it was possible (i.e., when more than one experiment on the same effect was reported in the paper in question). We believe that this is unfortunate, since this simple procedure can greatly reduce the uncertainty of the effect size estimation, providing more reliable estimates (Cumming, 2013). This, alongside the failure of most authors to interpret intervals, might constitute an important problem that should be addressed in future years.

Data on the effect size interpretation were also particularly interesting. We initially expected to find that authors tend not interpret effect sizes, whether they be standardized or unstandardized. However, we found a sharp improvement in this practice in the two journals. While this would appear to be a good sign, it might also reflect some issues related to the approach that we decided to use, which was very lenient. For this reason, we believe that this issue requires further research and using a more detailed coding.

Concerning Open Practices, we found large improvements in recent years. Our two top journals, at least, now tend to implement the open practices to a larger extent than in the past (Giofrè et al., 2017). Although there was a positive improvement in both journals, percentages were higher for PS than for JEPG. One reason for this could be the incentive of earning a badge for each open practice in PS, however, we can’t separate this effect from the influence of journal-specific submission guidelines in PS (Giofrè et al., 2017). In addition, research has shown that requiring authors to include a data availability statement can be extremely beneficial (e.g., Colavizza et al., 2020), and our results are consistent with this finding. It is also worth noting that in past years it was often hard to retrieve data and materials from online repositories (Giofrè et al., 2017), but we found that, over the past 6 years, data and materials are now relatively easy to access. For example, the majority of papers reporting open data now include that data on a reliable server (e.g., Open Science Framework); in only a few cases were we unable to retrieve the data (e.g., papers published in a personal or unreliable repository, authors making the content unavailable after the publication of the work).

Considering preregistered studies, we found that the percentage of these has considerably increased, having been extremely low in the past (see Giofrè et al., 2017). We found that about 20% in JEPG in 2020 were preregistered studies and almost twice that in PS, consistent with the availability of badges being a good strategy for boosting the use of this important practice. However, we did not assess the quality of preregistrations, although we noticed that some preregistrations provided only minimal information. For example, on AsPredicted.org (https://aspredicted.org/; accessed September 2021), preregistration requires authors only to answer a series of prompts, such as the following: hypothesis, dependent variable, conditions, analyses, outliers and exclusions, and sample size. Answering only a few basic such questions may result in a lack of completeness in the description of all methodological and statistical details, and so deviations from initial plans might not be identifiable. A full report should permit identification of deviation from what was preregistered. The problem is deviation in details that were not specified in a broad-brush preregistration is difficult to identify.

With this report, we also wanted to shed light on a new aspect, that is, the provision of statistical code. A growing number of authors are now making the code available. Doing so is a crucial way to facilitate the reproducibility of the results (Artner et al., 2021). It is also worth mentioning that percentages in PS, which strongly encourages provision of code, are considerably higher than in JEPG, which encourages open data and materials, but doesn’t mention code explicitly. We think that this is a very encouraging phenomenon that could probably be boosted, for example by providing a specific badge for this practice, or an annotation to the Open Data badge. This would probably also facilitate the access to this information.

To conclude, despite the adoption of some positive practices, we also found some important issues. For a start, confidence intervals are almost inevitably neglected, and many authors seem not to be aware of this problem. Reporting confidence intervals *per se* is probably insufficient if authors are not fully aware of problems related to the precision of their estimates, which inevitably linked to the issue of lack of statistical power in psychological research (Collins & Watt, 2021). We can speculate that substantial innovation and changes are probably not only reliant on journal practices and incentives, but also on a greater education about these practices. This goal can only be achieved if future generations of researchers are specifically trained in a better and more sensible way on these important aspects, otherwise changes might be only superficial.

### Supplementary information

ESM 1 (DOCX 254 kb)

## References

[CR1] Agnoli F, Wicherts JM, Veldkamp CLS, Albiero P, Cubelli R (2017). Questionable research practices among Italian research psychologists. PLOS ONE.

[CR2] American Psychological Association (2020). *Publication Manual of the American Psychological Association*.

[CR3] Artner R, Verliefde T, Steegen S, Gomes S, Traets F, Tuerlinckx F, Vanpaemel W (2021). The reproducibility of statistical results in psychological research: An investigation using unpublished raw data. Psychological Methods.

[CR4] Blanca MJ, Alarcón R, Bono R (2018). Current practices in data analysis procedures in psychology: What has changed?. Frontiers in Psychology.

[CR5] Blohowiak, B. B., Cohoon, J., de-Wit, L., Eich, E., Farach, F. J., Hasselman, F., ..., Lowrey, O. (2018). *Badges to acknowledge open practices*. Retrieved from http://www.osf.io/tvyxz

[CR6] Calin-Jageman RJ, Cumming G (2019). The new statistics for better science: Ask how much, how uncertain, and what else is known. The American Statistician.

[CR7] Camerer, C. F., Dreber, A., Holzmeister, F., Ho, T.-H., Huber, J., Johannesson, M., Kirchler, M., Nave, G., Nosek, B. A., Pfeiffer, T., Altmejd, A., Buttrick, N., Chan, T., Chen, Y., Forsell, E., Gampa, A., Heikensten, E., Hummer, L., Imai, T., Wu, H. (2018). Evaluating the replicability of social science experiments in Nature and Science between 2010 and 2015. *Nature Human Behaviour*, *2*(9), 637–644. 10.1038/s41562-018-0399-z 10.1038/s41562-018-0399-z 31346273

[CR8] Colavizza G, Hrynaszkiewicz I, Staden I, Whitaker K, McGillivray B (2020). The citation advantage of linking publications to research data. PLOS ONE.

[CR9] Collins E, Watt R (2021). Using and understanding power in psychological research: A survey study. Collabra: Psychology.

[CR10] Cumming G (2013). The new statistics: A how-to guide. Australian Psychologist.

[CR11] Cumming G (2014). The new statistics: Why and how. Psychological Science.

[CR12] Giofrè D, Cumming G, Fresc L, Boedker I, Tressoldi P (2017). The influence of journal submission guidelines on authors’ reporting of statistics and use of open research practices. PLOS ONE.

[CR13] Goldacre B, Morton CE, DeVito NJ (2019). Why researchers should share their analytic code. British Medical Journal.

[CR14] Goodman SN, Fanelli D, Ioannidis JPA (2016). What does research reproducibility mean?. Science Translational Medicine.

[CR15] Houtkoop BL, Chambers C, Macleod M, Bishop DVM, Nichols TE, Wagenmakers E-J (2018). Data sharing in psychology: A survey on barriers and preconditions. Advances in Methods and Practices in Psychological Science.

[CR16] Kidwell MC, Lazarević LB, Baranski E, Hardwicke TE, Piechowski S, Falkenberg L-S, Kennett C, Slowik A, Sonnleitner C, Hess-Holden C, Errington TM, Fiedler S, Nosek BA (2016). Badges to acknowledge open practices: A simple, low-cost, effective method for increasing transparency. PLOS Biology.

[CR17] Lindsay DS (2015). Replication in psychological science. Psychological Science.

[CR18] Nosek, B. A., Alter, G., Banks, G. C., Borsboom, D., Bowman, S. D., Breckler, S. J., Buck, S., Chambers, C. D., Chin, G., Christensen, G., Contestabile, M., Dafoe, A., Eich, E., Freese, J., Glennerster, R., Goroff, D., Green, D. P., Hesse, B., Humphreys, M., Yarkoni, T. (2015). Promoting an open research culture. *Science*, *348*(6242), 1422–1425. 10.1126/science.aab2374 10.1126/science.aab2374 PMC4550299 26113702

[CR19] Nosek, B. A., Ebersole, C. R., DeHaven, A. C., & Mellor, D. T. (2018). Reply to Ledgerwood: Predictions without analysis plans are inert. *Proceedings of the National Academy of Sciences, 115*(45). 10.1073/pnas.1816418115 10.1073/pnas.1816418115 PMC6233068 30341224

[CR20] Open Science Collaboration (2015). Estimating the reproducibility of psychological science. Science.

[CR21] Szucs D, Ioannidis JPA (2017). Empirical assessment of published effect sizes and power in the recent cognitive neuroscience and psychology literature. PLOS Biology.

[CR22] Tressoldi PE, Giofrè D (2015). The pervasive avoidance of prospective statistical power: Major consequences and practical solutions. Frontiers in Psychology.

[CR23] Tressoldi PE, Giofrè D, Sella F, Cumming G (2013). High Impact = High Statistical Standards? Not necessarily so. PLoS ONE.

