# Supplementary material for: The influence of journal submission guidelines on authors’ reporting of statistics and use of open research practices: Five years later
Source: Behav Res Methods. 2022 Oct 17;55(7):3845–54. doi: 10.3758/s13428-022-01993-3 (PMC10615932; doi:10.3758/s13428-022-01993-3)

Supplementary online materials

**Figure S1.**

*Percentage of papers using NHST from 2013 to 2020 in Psychological Science and in the Journal of Experimental Psychology: General*


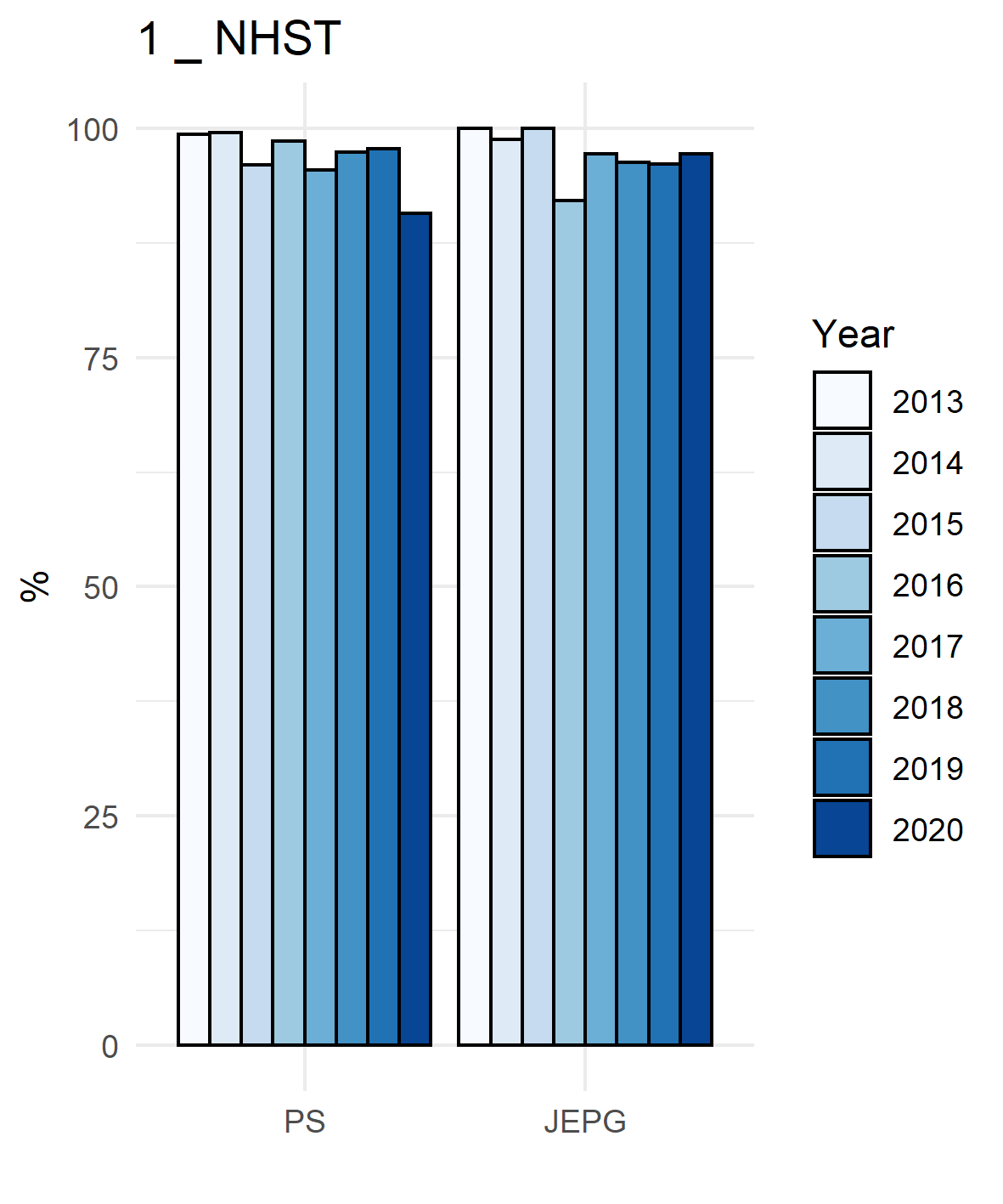


**Figure S2.**

*Percentage of papers using CI from 2013 to 2020 in Psychological Science and in the Journal of Experimental Psychology: General.*


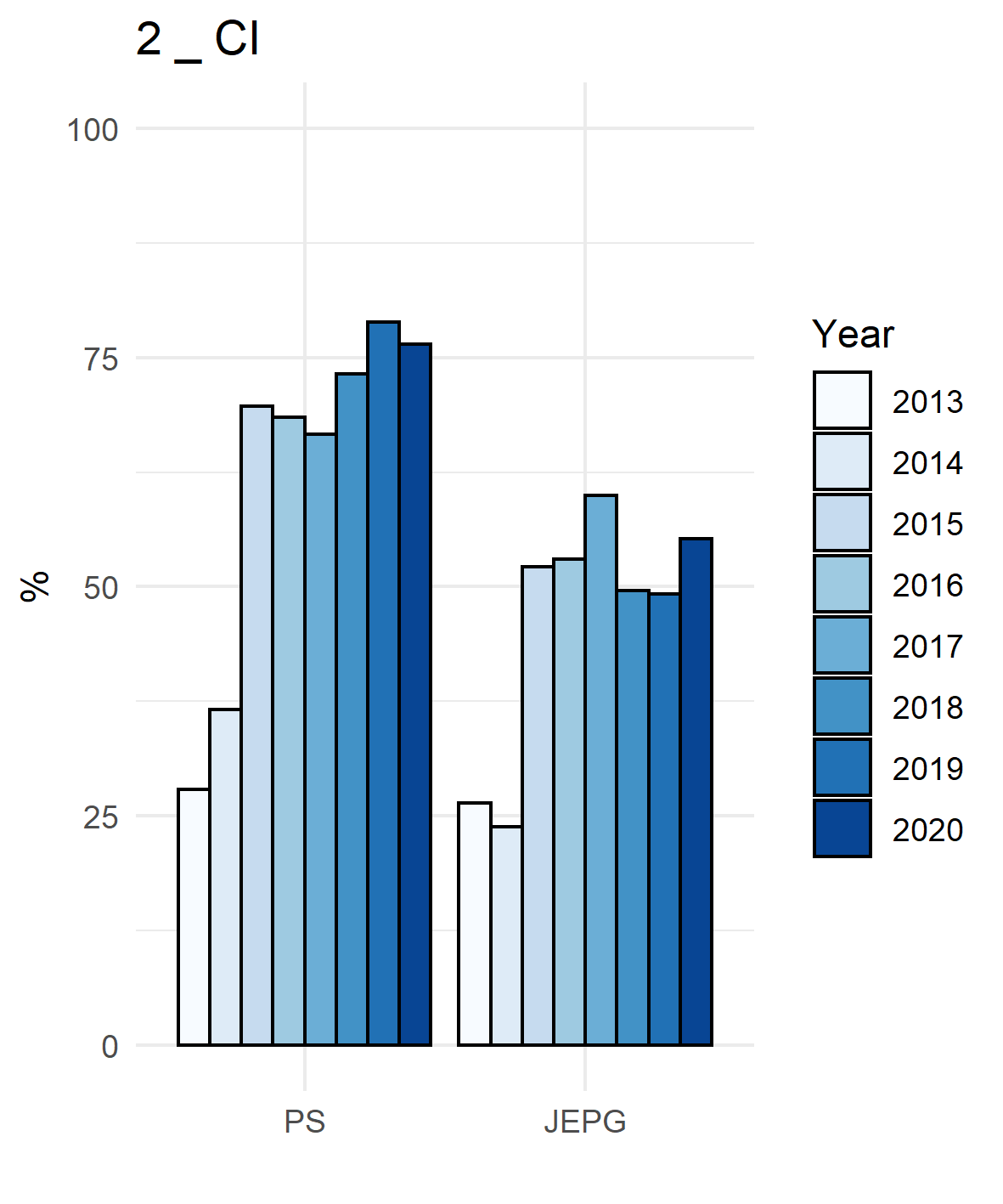


**Figure S3.**

*Percentage of papers using meta-analysis from 2013 to 2020 in Psychological Science and in the Journal of Experimental Psychology: General.*


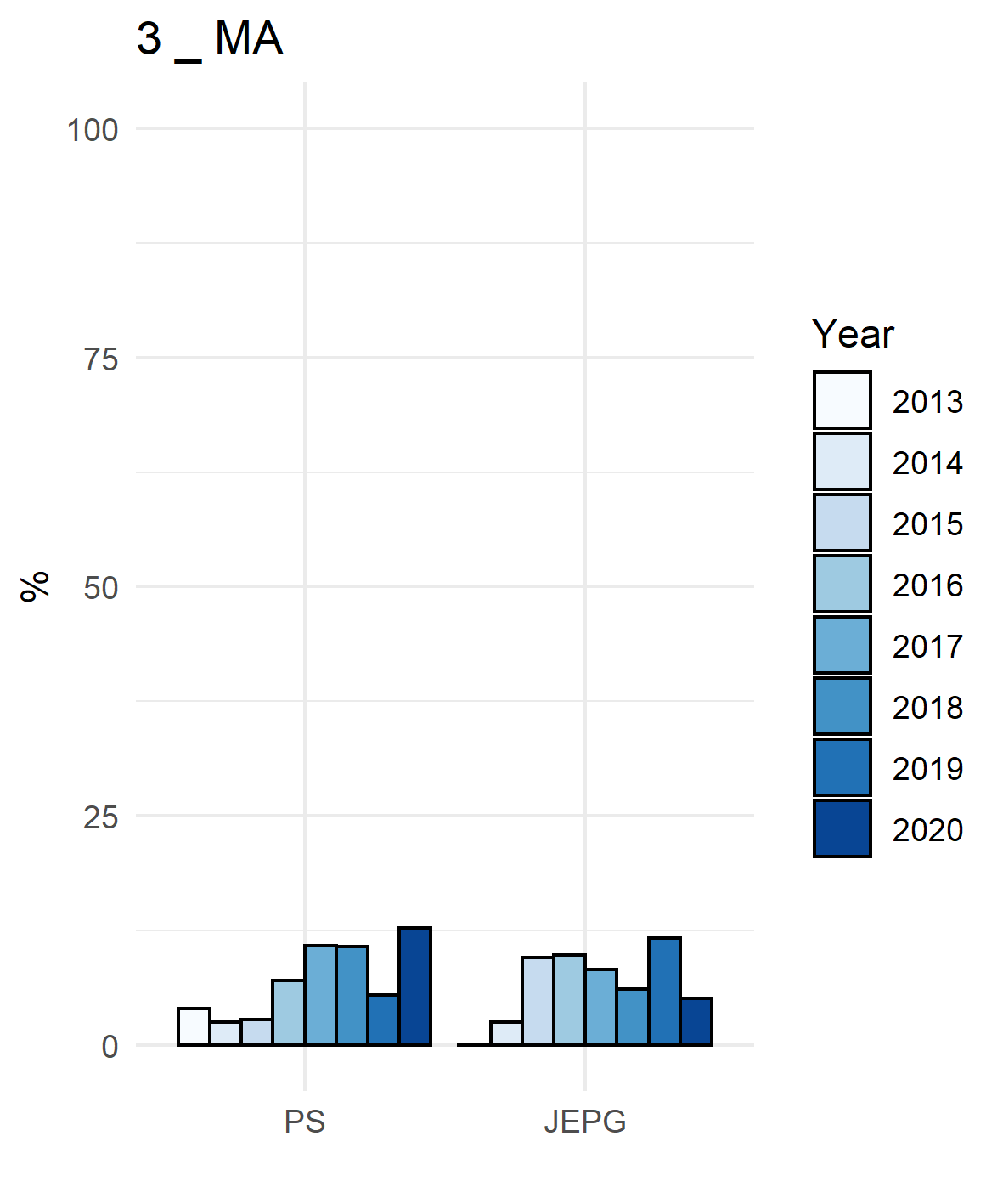


**Figure S4.**

*Percentage of papers using confidence intervals interpretation from 2013 to 2020 in Psychological Science and in the Journal of Experimental Psychology: General*


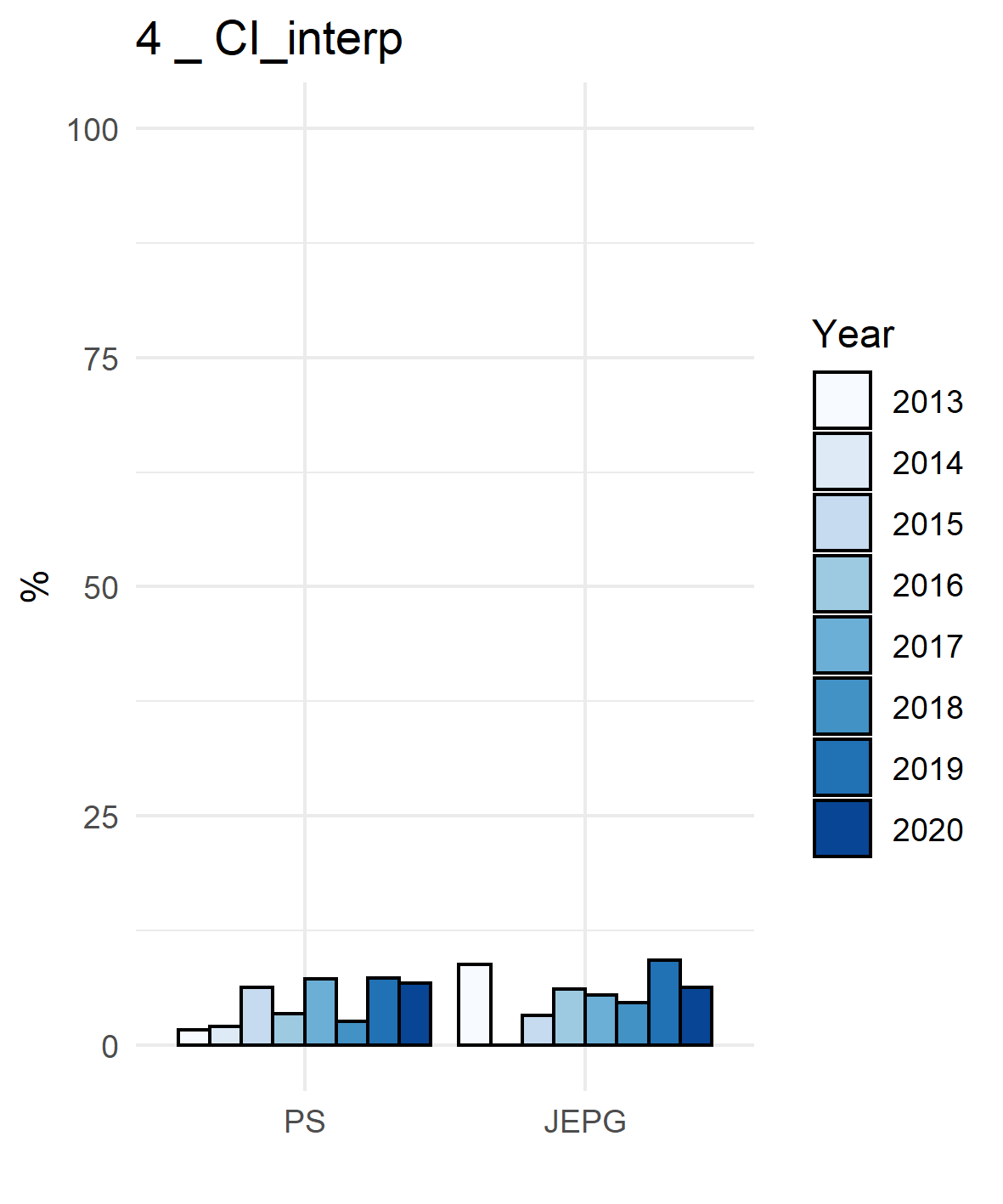


**Figure S5.**

*Percentage of papers reporting effect size interpretation from 2013 to 2020 in Psychological Science and in the Journal of Experimental Psychology: General.*


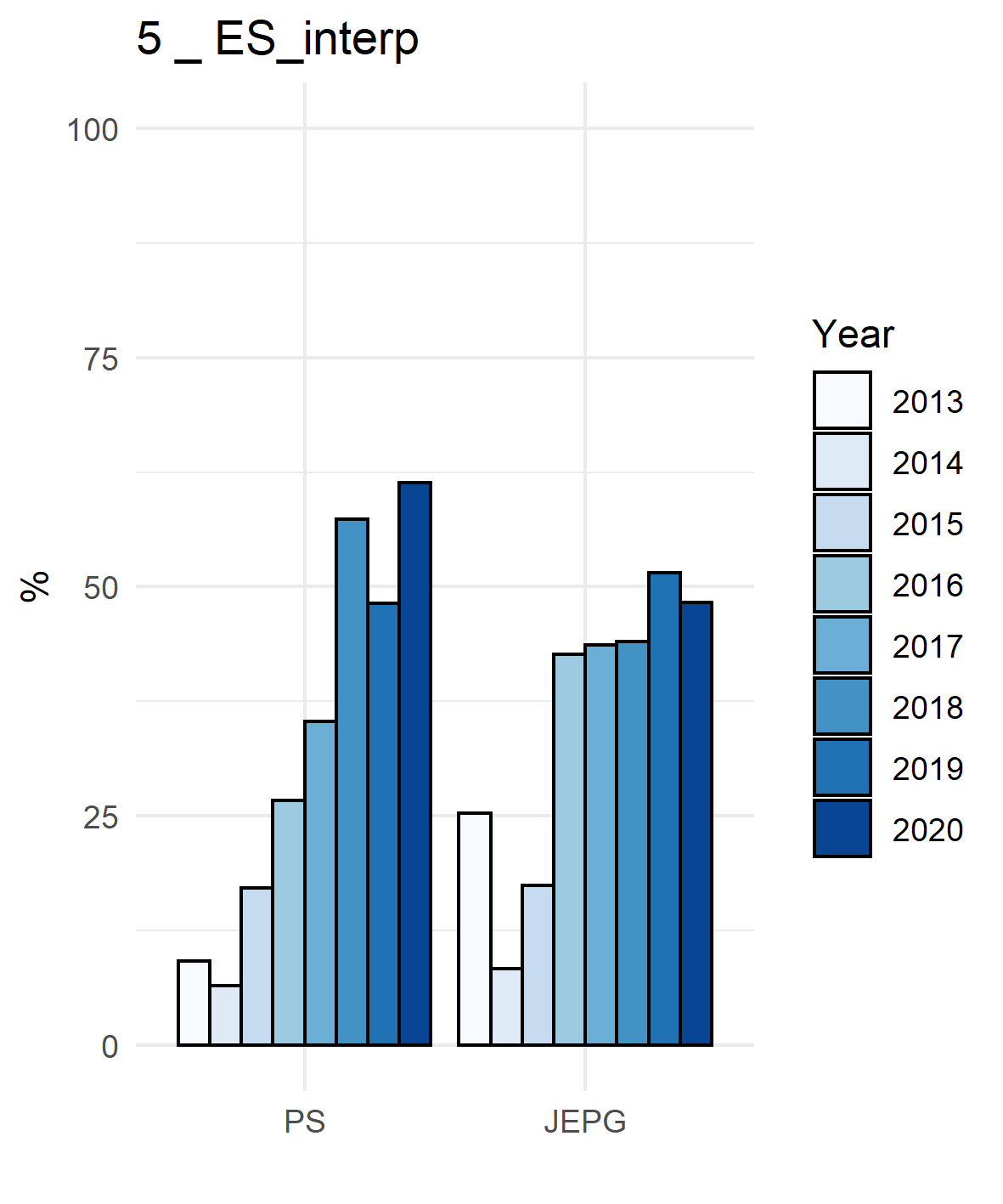


**Figure S6.**

*Percentage of papers using information about sample size determination from 2013 to 2020 in Psychological Science and in the Journal of Experimental Psychology: General.*


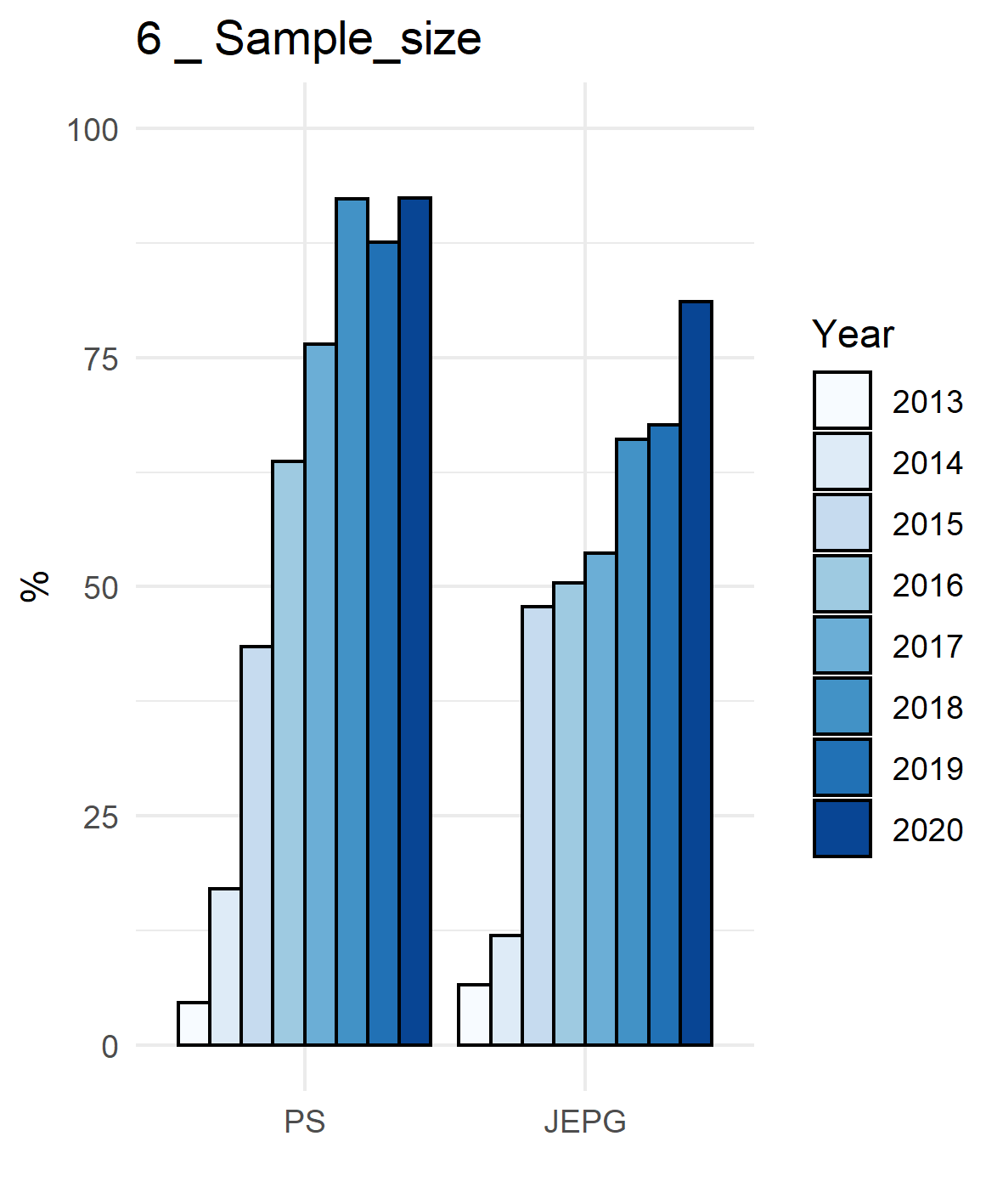


**Figure S7.**

*Percentage of papers reportingdata exclusion from 2013 to 2020 in Psychological Science and in the Journal of Experimental Psychology: General.*


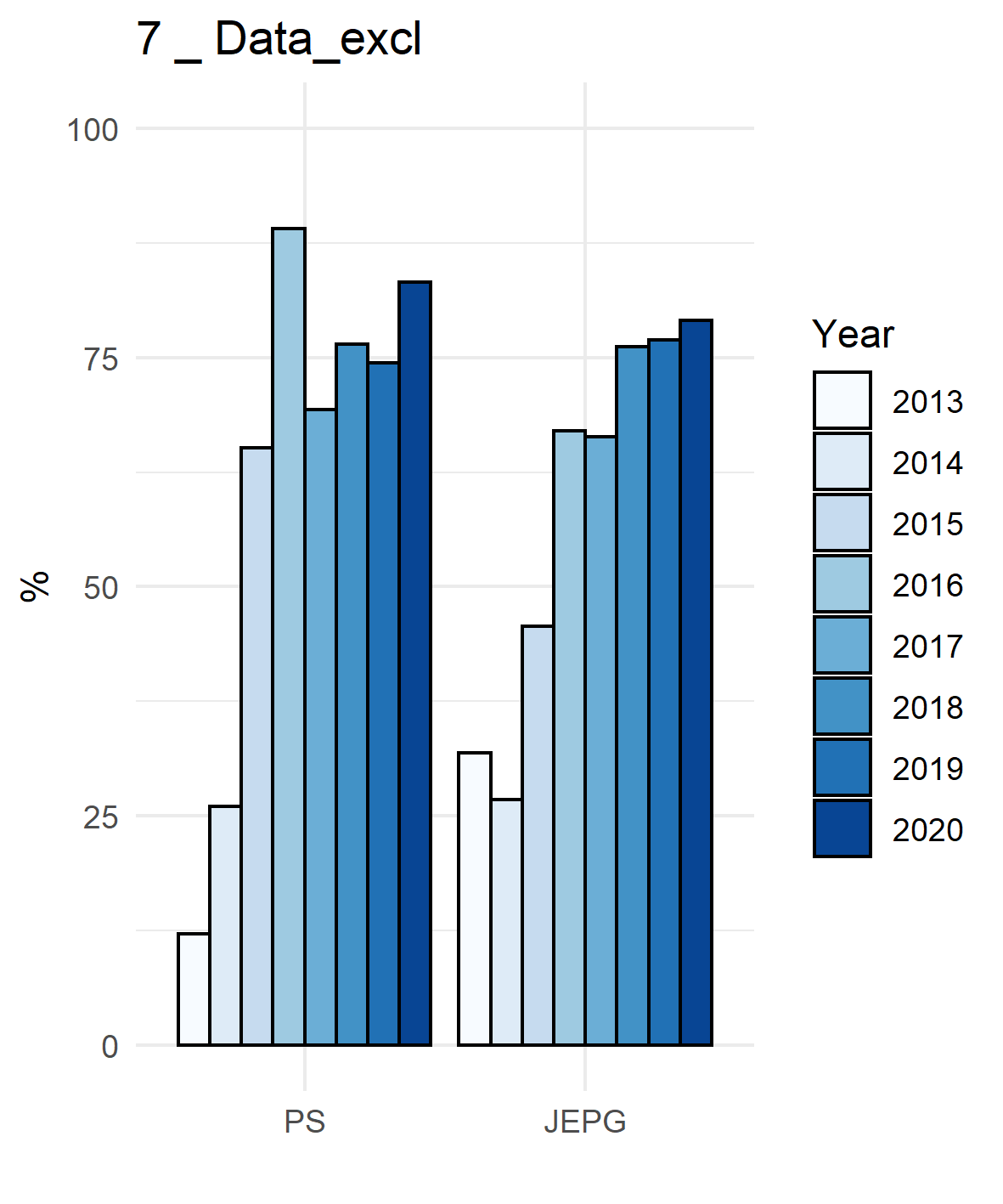


**Figure S8.**

*Percentage of papers reporting data availability from 2013 to 2020 in Psychological Science and in the Journal of Experimental Psychology: General.*


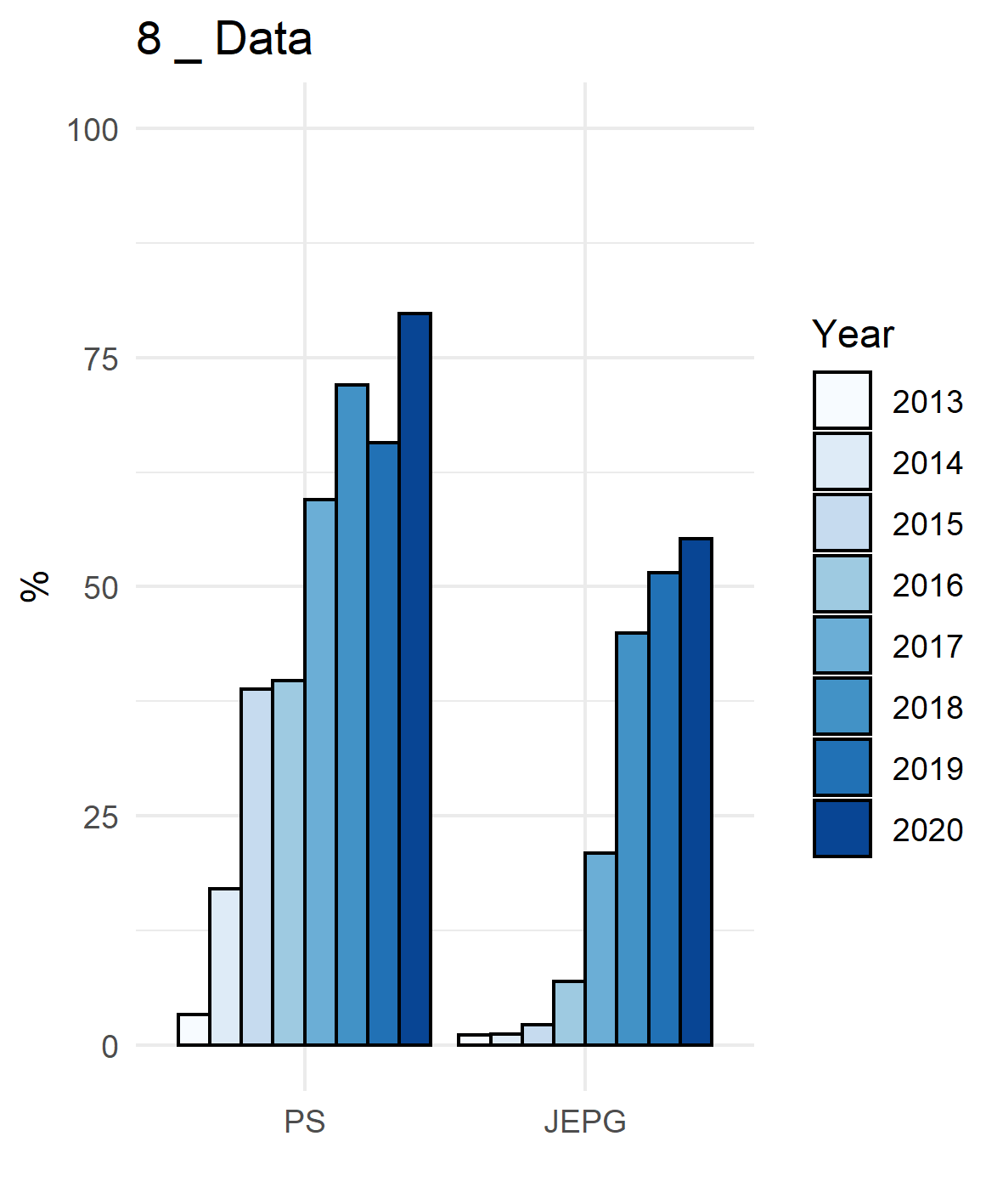


**Figure S9.**

*Percentage of papers reporting materials availability from 2013 to 2020 in Psychological Science and in the Journal of Experimental Psychology: General.*


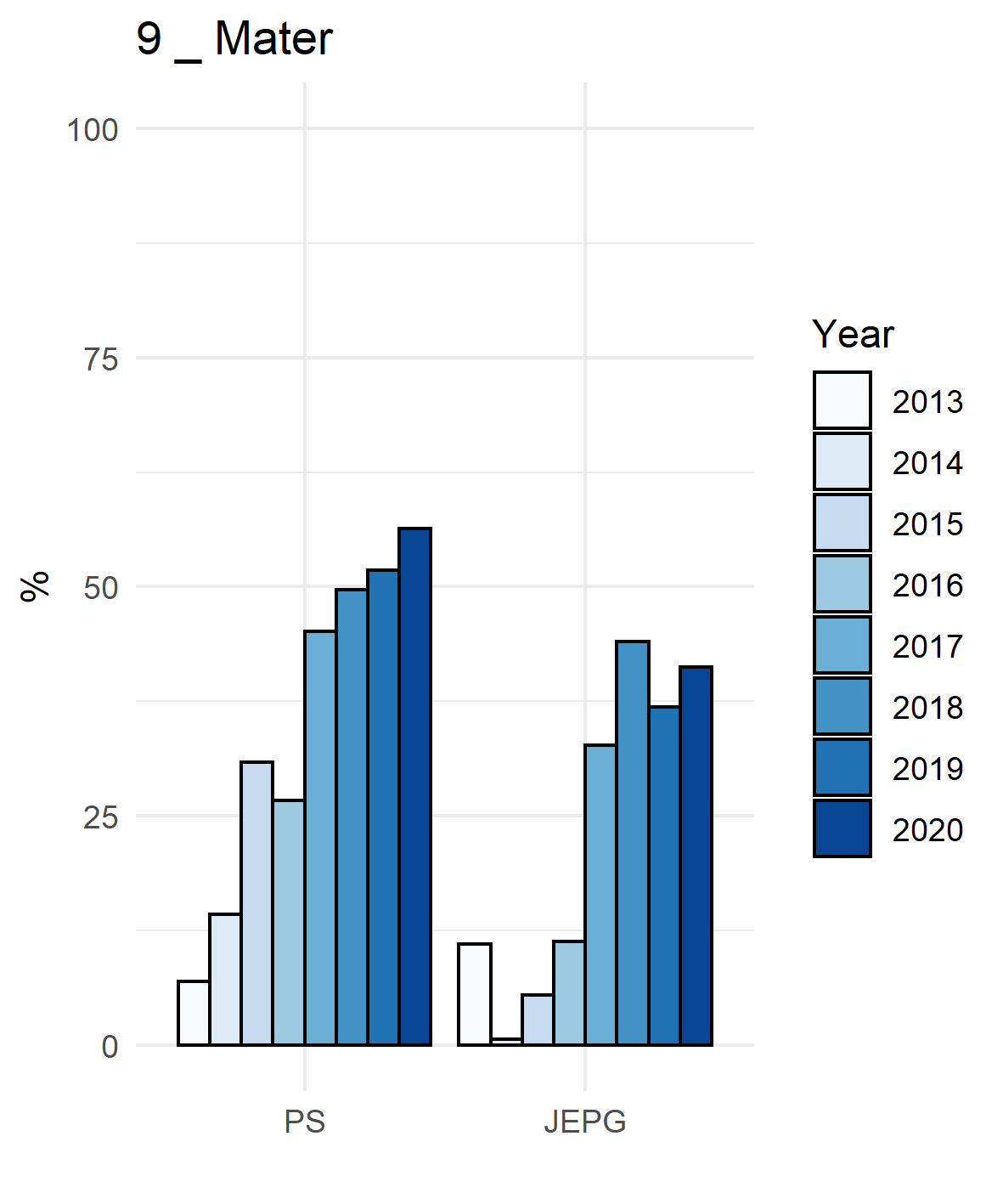


**Figure S10.**

*Percentage of papers reporting presence of a preregistration from 2013 to 2020 in Psychological Science and in the Journal of Experimental Psychology: General.*


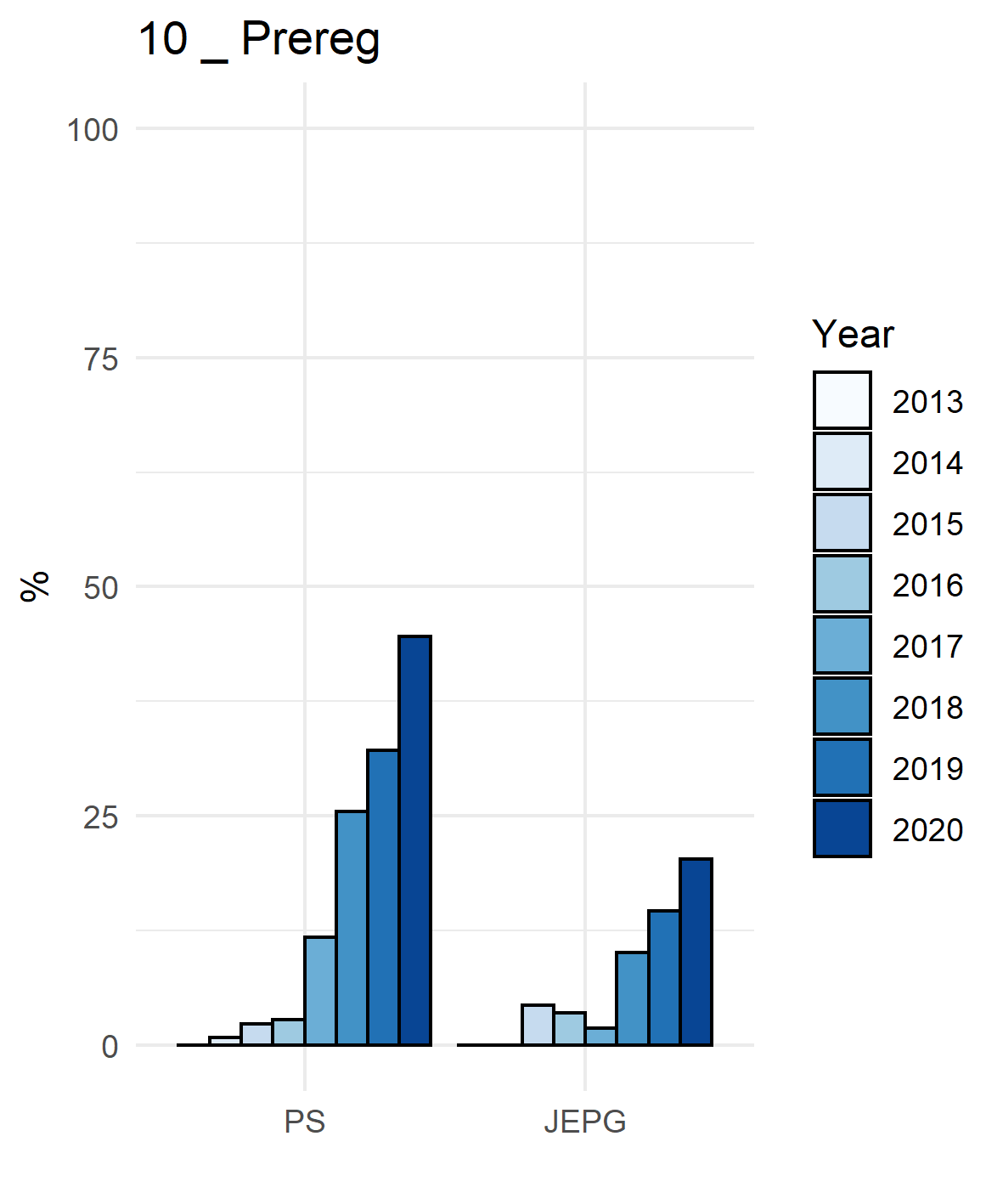


**Figure S11.**

*Percentage of papers reporting presence of the code from 2016 to 2020 in Psychological Science and in the Journal of Experimental Psychology: General.*


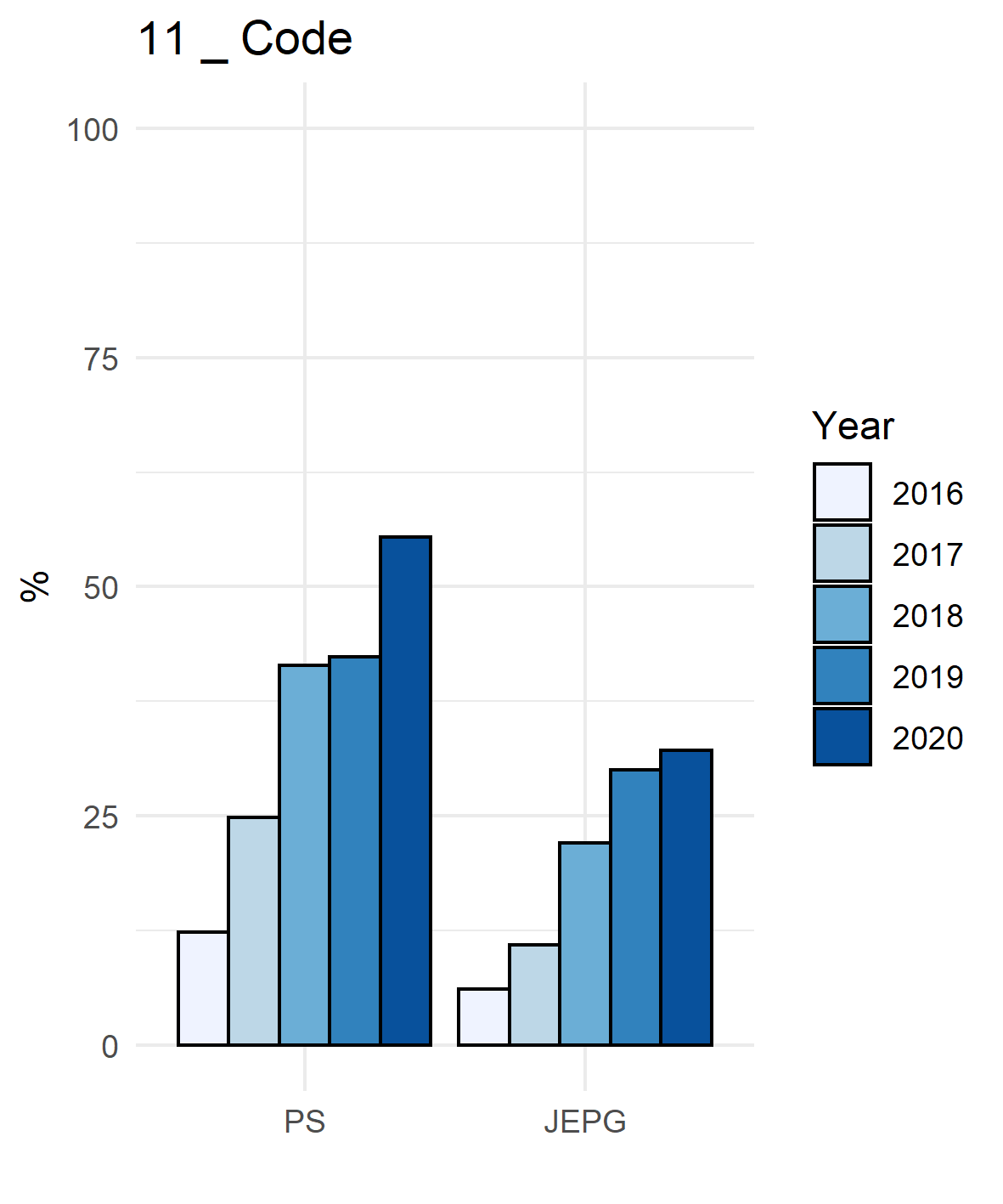

Supplement: Supplementary file 1 — (DOCX 254 kb) [file 13428_2022_1993_MOESM1_ESM.docx]
